# Novel in-frame duplication variant characterization in late infantile metachromatic leukodystrophy using whole-exome sequencing and molecular dynamics simulation

**DOI:** 10.1371/journal.pone.0282304

**Published:** 2023-02-27

**Authors:** Zahra Ataei, Zahra Nouri, Farial Tavakoli, Mohammad Reza Pourreza, Sina Narrei, Mohammad Amin Tabatabaiefar

**Affiliations:** 1 Department of Genetics and Molecular Biology, School of Medicine, Isfahan University of Medical Sciences, Isfahan, Iran; 2 Pediatric Inherited Diseases Research Center, Research Institute for Primordial Prevention of Noncommunicable Disease, Isfahan University of Medical Sciences, Isfahan, Iran; 3 Department of Cell and Molecular Biology & Microbiology, Faculty of Biological Science and Technology, University of Isfahan, Isfahan, Iran; 4 Core research facilities, Isfahan University of Medical Sciences, Isfahan, Iran; 5 Department of Research and Development, ERYTHROGEN Medical Genetics Lab, Isfahan, Iran; National Institute for Biotechnology and Genetic Engeneering, PAKISTAN

## Abstract

Metachromatic leukodystrophy (MLD) is a neurodegenerative lysosomal storage disease caused by a deficiency in the arylsulfatase A (ARSA). ARSA deficiency leads to sulfatide accumulation, which involves progressive demyelination. The profound impact of early diagnosis on MLD treatment options necessitates the development of new or updated analysis tools and approaches. In this study, to identify the genetic etiology in a proband from a consanguineous family with MLD presentation and low ARSA activity, we employed Whole-Exome Sequencing (WES) followed by co-segregation analysis using Sanger sequencing. Also, MD simulation was utilized to study how the variant alters the structural behavior and function of the ARSA protein. GROMACS was applied and the data was analyzed by RMSD, RMSF, Rg, SASA, HB, atomic distance, PCA, and FEL. Variant interpretation was done based on the American College of Medical Genetics and Genomics (ACMG) guidelines. WES results showed a novel homozygous insertion mutation, c.109_126dup (p.Asp37_Gly42dup), in the *ARSA* gene. This variant is located in the first exon of *ARSA*, fulfilling the criteria of being categorized as likely pathogenic, according to the ACMG guidelines and it was also found to be co-segregating in the family. The MD simulation analysis revealed this mutation influenced the structure and the stabilization of ARSA and led to the protein function impairment. Here, we report a useful application of WES and MD to identify the causes of a neurometabolic disorder.

## Introduction

Leukodystrophies are a group of usually inherited disorders that affect the white matter of the central nervous system [1]. Metachromatic leukodystrophy (MLD) (OMIM 250100), as an autosomal recessive neurodegenerative disorder, is one of the most common leukodystrophies. MLD is one of the lysosomal storage diseases (LSDs) with a prevalence of 1.45 per 100,000 births worldwide [2]. MLD is a sphingolipidosis caused by lysosomal enzyme arylsulfatase A (ARSA**)** deficiency or its sphingolipid activator protein B (SapB) [3]. The *ARSA* gene is located on chromosome 22q13 with eight exons spanning a genomic region of 3kb and encoding a 509 amino acids [4, 5] ARSA is responsible for the hydrolysis of the 3-O ester bond of sphingolipid 3′-O-sulfogalactosylceramide, known as sulfatide. ARSA deficiency leads to an increase in the sulfatide within oligodendrocytes, macrophages, and some subtypes of neurons in the CNS, in Schwann cells, and in the peripheral nervous system (PNS) macrophages, which exhibit metachromatic staining characteristics [6]. Instability of the myelin sheath, change in calcium homeostasis, cell stress, apoptosis, and inflammatory response are consequences of sulfatide deposition in the cell cytoplasm [1, 7].

Depending upon the age which symptoms present, MLD is classified into three different clinical forms: late infantile, juvenile, and adult forms [8]. Late-infantile MLD, the most severe type with poor prognosis, is characterized by gait abnormalities, seizures, ataxia, hypotonia, extensor planters, optic atrophy, and regression of motor skills that lead to complete gross motor deterioration before the age of 40 months [9, 10].

There is no newborn screening test available for MLD diagnosis, and MLD is diagnosed after birth [11]. Progressive demyelination and subsequent neurological symptoms, biochemical procedures, genetic analysis, and imaging results should be applied for a specific diagnosis of MLD. Biochemical assays, including the quantification of Sulfatide accumulation in urine, and ARSA activity in peripheral blood leukocytes, are used for accurate diagnosis of MLD [12].

Next generation sequencing techniques provide an opportunity for diagnosis of hereditary genetic diseases in research and clinical settings. Whole exome sequencing (WES) has been applied in identifying genetic variants associated with a variety of diseases [13, 14]. Moreover, the biocomputational techniques have been recently applied broadly as a potential tool to investigate the variant effect on a mutant protein structure, in a rapid and cost-effective manner [15, 16]

In the current study, for accurate diagnosis of a 34- month patient, the first child of consanguineous parents with a progressive decline of motor and cognitive abilities, hypotonia, and spasticity, we used WES in the diagnosis of MLD, followed by imaging analysis and enzymatic tests aimed at confirming the molecular results, which led to a novel in-frame mutation identification. Furthermore, Molecular dynamics (MD) simulation was applied to investigate the ARSA in wild type form (WT-ARSA) and structural and conformational changes of mutant form of ARSA (mutant-ARSA) to understand the pathogenic mechanism of MLD disease at atomic level.

## Material and methods

### Subject and clinical evaluations and imaging results

The study was approved by the Research Ethics Committee of “Alzahra Research Center” (grant no:2400173, IR.ARI.MUI.REC.1400.011), and the patient and his parents were recruited to the study after obtaining informed consent. The proband was a 34-month boy and the first child of consanguineous Iranian parents who were first cousins without a family history of neurological disease. A detailed clinical examination and comprehensive family history were done by a clinical geneticist.

### ARSA enzymatic assay

ARSA activity was estimated as mu/mg protein in leukocytes, using 4-nitrocatechol sulfate. In brief: 0.25 mM sodium pyrophosphate was utilized to inactivate arylsulfatase B(ARSB). Then, the amount of sulfate released was measured by the absorbance of free 4-nitrocatechol at 515 nm on a spectrophotometer (Beckmann Coulter, Brea, CA, USA), which is associated with sulfatase activity [17].

### Metabolic panel test

Amino Acid Profile and AcylCarnitine Profile were investigated in plasma using LC-MS/MS and MS/MS, respectively. Also, Organic Acids and Acylglycines measurements in urine were performed through GC-MS and LC-MS/MS.

### Whole exome sequencing

Blood samples were taken from the patient and his parents. Using a Qiagen DNA extraction kit, genomic DNA was extracted from peripheral blood lymphocytes, and assessment of its purity, was done on a Nanospec Cube Biophotometer (Nanolytik®, Dusseldorf, Germany). The sample was sent to Macrogen (South Korea) (https://www.macrogen.com/) for WES analysis using the Novaseq 4000 platform (Illumina, San Diego, CA, USA) with the mean depth of coverage 100X. These samples were sheared into 151-bp fragments by a hydrodynamic shearing system (Covaris, Massachusetts, USA), and whole exome was captured through in-solution targeted genomic enrichment using Agilent SureSelect Human All Exon kit v6 (Agilent Technologies, CA, USA).

### Bioinformatics analysis

Following sequencing, image analysis and base-calling were performed using the standard Illumina data analysis pipeline Real-Time Analysis (RTA) version (RTA) v1.12.4. CASAVA v1.8.2. The raw reads quality was assessed using the FastQC [18]. The low quality reads have been removed with TRIMMOMATIC [19] and quality control was done after trimming. Reads were mapped to the human reference genome build UCSC hg19 (http://genome.ucsc.edu/) using BWA (Burrows-Wheeler Aligner) (http://bio-bwa.sourceforge.net/). SAMtools was used to convert sequence alignment map (SAM) format to sorted, indexed binary alignment map files [20]. GATK software tools (https://gatk.broadinstitute.org/hc/en-us) were used to improve alignments and genotype calling. annotation was performed using ANNOVAR [21].

Missense, nonsense, start codon change, stop loss, indel variants, and splice site with Minor Allele Frequency <1% in dbSNP version 147, 1000 genomes project phase 3 database(https://www.internationalgenome.org/), NHLBI GO exome sequencing project (ESP) (https://evs.gs.washington.edu/EVS/), exome aggregation consortium (ExAC) (https://exac.broadinstitute.org/), Iranome database (http://www.iranome.ir/), and our locally developed database (GTAC) are considered for further analysis. The novelty of the variant was investigated in the Human Gene Mutation Database (HGMD) (http://www.hgmd.cf.ac.uk/ac/index.php) and the literature. Finally, the identified variants’ pathogenicity can be interpreted according to ACMG 2015 standards and guidelines for interpreting sequence variants [22]. The MEGA6 software was utilized to investigate the conservation of the mutated region among several species [23].

### Variant confirmation

The candidate variant (p.Asp37_Gly42dup) that was identified by WES was subsequently confirmed using polymerase chain reaction (PCR) and bidirectional Sanger sequencing in the proband Using the SeqStudio Genetic Analyzer (Applied Biosystem Inc., Foster City, CA, USA). Then, to examine the segregation of genotype among the family members, co-segregation analysis was performed on his parents. Primers (forward primer: 5′- GTATTTGGGTCCGGGGTCTC-3′ and the reverse primer: 5′-TGTGGCCTTCCCTAGAGAGA-3′ (designed by Primer 3 software Input version 0.4.0 (https://primer3.ut.ee/) and NCBI primer BLAST software)) encompassed exon 1 of the *ARSA* gene. Chromatogram sequences files were compared with the reference sequence (NM_000487), via SeqMan software version 5.00© (DNASTAR, Madison, WI, USA).

### Molecular modeling

Crystal structure of WT-ARSA protein in complex with NAG1 and NAG2 was obtained from Protein Data Bank (PDB ID: 1AUK with resolution 2.10 Å). Only protein molecule was retained and all excess molecules were removed from the complex using Discovery Studio 2016 Visualizer software (DS 2016) (DS 4.0, Accelrys Software Inc., San Diego, CA). Swiss Model webserver [24] was applied in order to repair the chain breaks in the WT-ARSA and construct a model for the mutant-ARSA. The modeled WT-ARSA is composed of 485 residues starting with residue Arg1 and ending with residue Pro485 while the modeled mutant-ARSA is composed of 491 residues containing the six-amino acid insertion mutation in 23–28 position (Asp23, Leu24, Gly25, Cys26, Tyr27, and Gly28) starting with residue Arg1 and ending with residue Pro491. The amino acid compositions of the active site of WT-ARSA are Ala10, Asp11, Asp12, FGly51, Arg55, Lys105, His107, His211, Asp263, Asn264, Lys284 and those of mutant-ARSA are Ala10, Asp11, Asp12, FGly57, Arg61, Lys111, His113, His217, Asp269, Asn270, and Lys290.

### Molecular dynamics simulation

In order to investigate the structural and conformational changes in ARSA proteins, GROMACS 2018 package was applied to perform molecular dynamics simulation [25]. PDB2PQR web server [26] was retrieved to determine the protonation state (PH = 5) of His residues in both proteins. All the required files, topology and coordinate files, for both molecules were constructed using GROMACS [25] through CHARMM27 all-atom force field [27].

A dodecahedron TIP3P water box with a direction of 9 Å as an unit cell was constructed to solvate all models [28]. In order to calculate each system in water, the neutralization of the negative charges of the system was carried out through adding Na+ ions. Energy-minimization of the solvated system was performed applying 50000 steps of steepest-descent method to remove steric clashes. Equilibration of the minimized systems with position restrain on the proteins were performed by NVT and NPT ensembles for 400 ps at a temperature of 300 K and pressure of 1 bar, respectively.

V-rescale temperature [29] and Parrinello-Rahman pressure [30] coupling methods were applied to stabilize the temperature and pressure at 300 K and 1 bar for the system. 100-ns MD simulations were carried out for ARSA proteins under periodic boundary condition with the time step of 2 fs applying LINCS [31] and Partial Mesh Ewald (PME) [32] algorithms. The coulomb and van der Waals interactions were calculated by cut-off value of 1.2 nm.

### Analysis of MD simulations

Production simulations were analyzed applying trajectory analysis modules in the GROMACS simulation package and visualized using VMD [33], Root mean square deviation (RMSD), root mean square fluctuation (RMSF), radius of gyration (Rg), solvent accessible surface area (SASA), hydrogen bond (HB), secondary structure, atomic distance, principal component analysis (PCA) and gibbs free energy landscape (FEL) were obtained using GROMACS and Grace-5.1.22/QTGrace v0.2.6 program was retrieved to visualize all the graphs.

#### Principal component analysis

Principal component analysis (PCA) was calculated to investigate the biomolecules motion [34] applying calculating the covariance matrix C:

Cij=<(xi−<xi>)(xj−<xj>)> (1)

Where *xi* and *xj* are the coordinate of the *ith* and *jth* atoms of the systems and <*xi*> and <*xj*> represent the average coordinate of the *ith* and *jth* atoms over the ensemble. Then, the principal components (PCs) are calculated by diagonalization of covariance matrix.

#### Free energy landscape

Free energy landscape (FEL) was calculated to investigate the conformational changes of biomolecules to identify the stable state and transient state of proteins and to assign their stability and their function [35]. The FEL can be calculated as:

ΔG(X)=−KBTlnP(X) (2)

Where ΔG (X) is the free energy, K_B_ and T, and P(X) represent the Boltzman constant, absolute temperature, and the probability distribution of the conformation ensemble along the PCs.

## Results

### Clinical evaluations

The proband was a 34-month boy and the first child of consanguineous Iranian parents without a family history of neurological disease. The function of kidneys, liver, thyroid, and heart was normal. He was born at full term via normal vaginal delivery after an uncomplicated pregnancy.

Prenatal screening tests did not show any abnormalities in the fetus. At the age of 15 months, he presented with spastic gait, and he could not walk independently. However, MRI results suggested a normal brain. Clonazepam was administered orally until two years, and manifestations of spasticity were controlled. The patient had no swallowing difficulties. At 32 months, the child had language disorder, weakness started in the hands, muscle spasms progressed, and muscular hypotonia was evident. Electroencephalogram (EEG) showed mildly abnormal, and MRI results show evidence of bilateral symmetrical abnormal signal intensity in deep white matter, centrum semiovale, paraventricular regions, and subcortical region as low signal intensity in T1, high signal intensity in T2W and flair images.

### ARSA enzyme activity

The enzyme activity using the colorimetric method was 0.066 mu/mg protein. The reference was 0.37–1.815.

### Metabolic panel

There was no abnormality in the metabolic profile of the patient in the mentioned factors.

### Molecular findings

**Fig 1:**
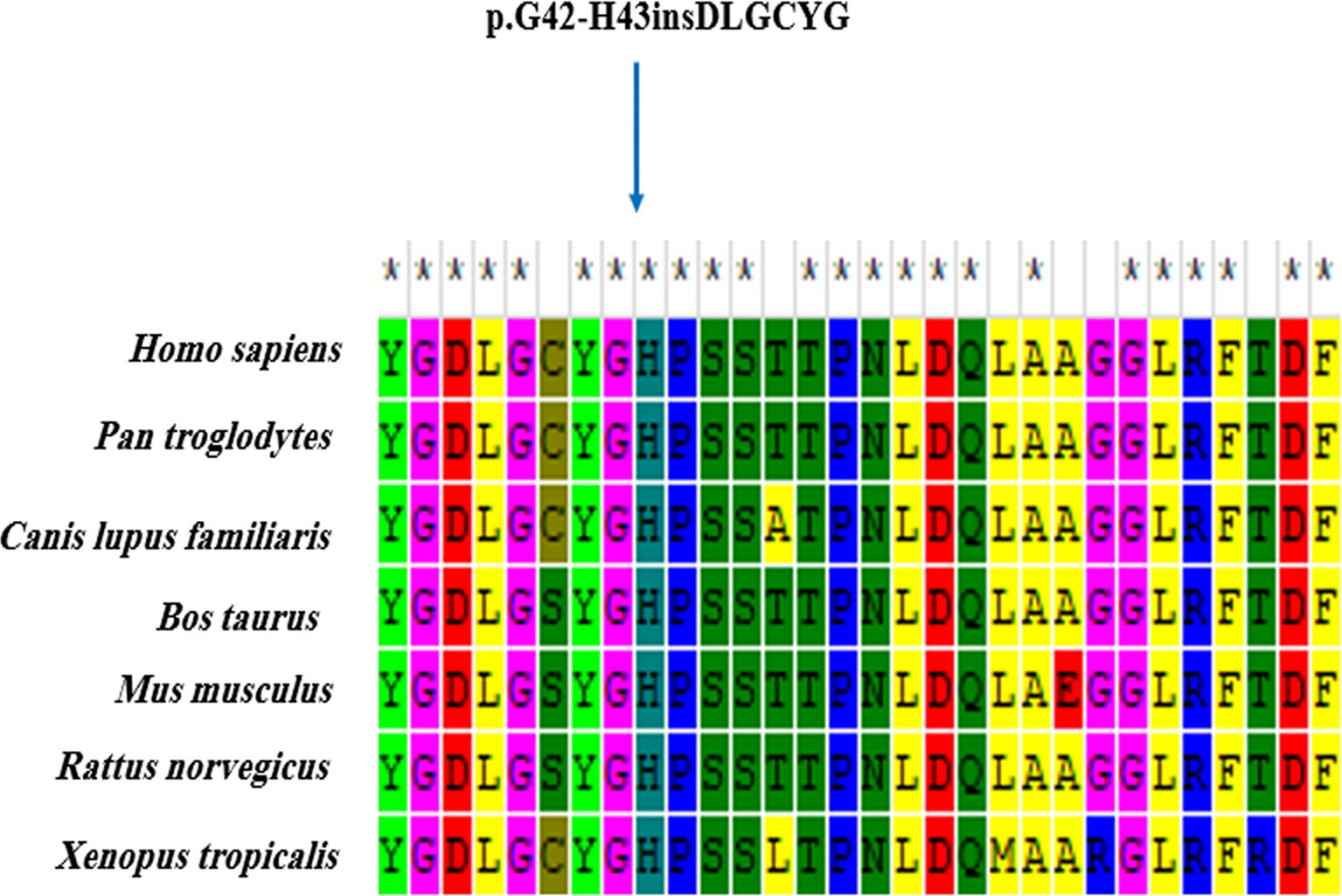
Phylogenetic alignment performed with MEGA6. The modified region is located in a highly conserved region among species.

**Fig 2:**
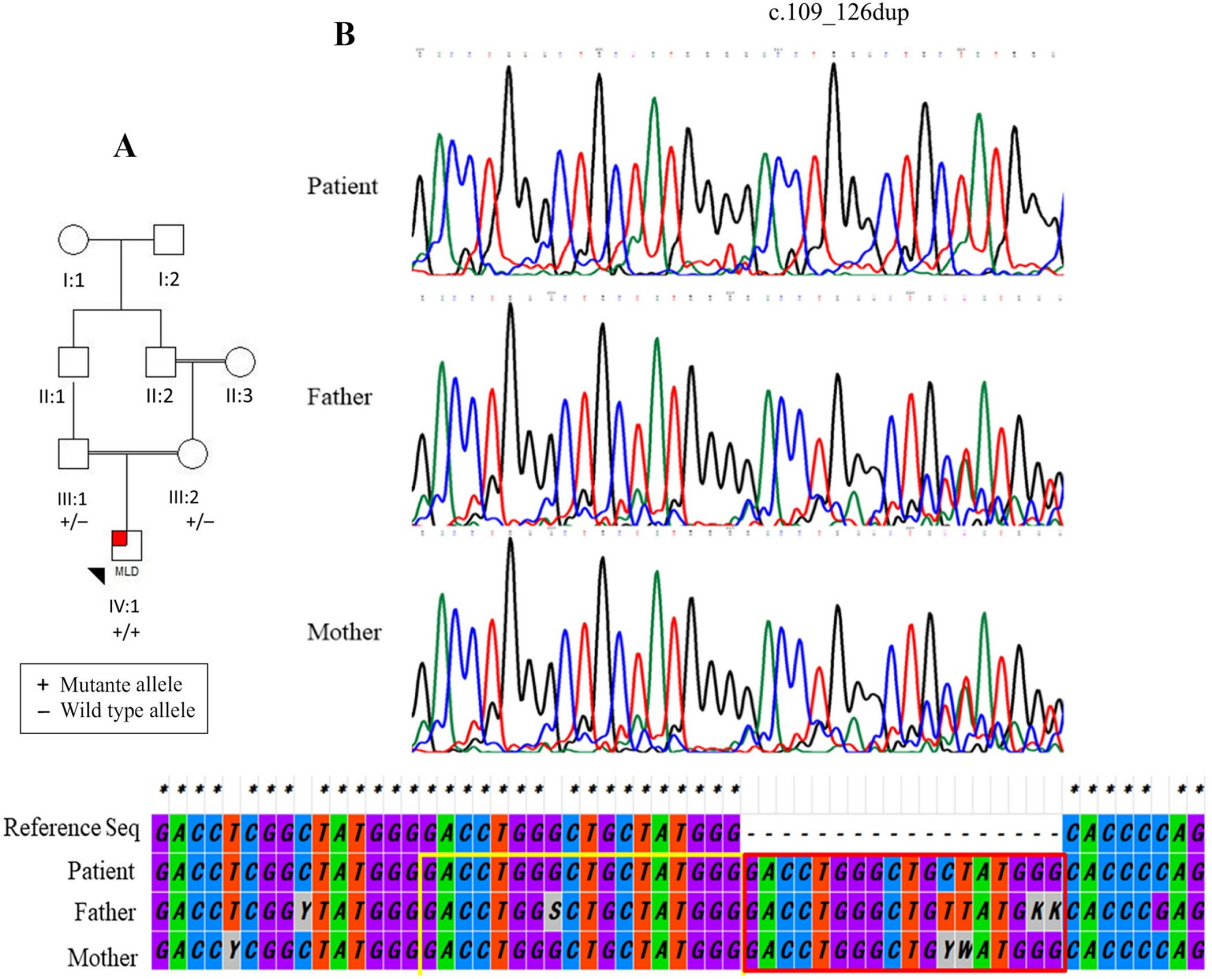
(A) Pedigree chart of the family. (B) Chromatogram of the homozygous sequence variant c.109_126dup detected in the *ARSA* gene in the proband compared with the heterozygous sequence in the parents. The comparison of three sequences with the reference sequence is given at the bottom of the electropherograms. The red and yellow boxes indicate the duplicated and the previous sequences, respectively.

Upon exome analysis, a total number of 28135 variants was obtained. The number of variants was limited by applying the following criteria for variant filtering, and a homozygous insertion of 18 nucleotides (**c.109_126dup)** in the *ARSA* gene was found. It causes in-frame insertion in exon 1 of this gene, resulting in a protein with 515 residues (versus 509 residues in the intact protein). The in-frame variant was absent from dbSNP version 147, 1000 genomes project phase 3, NHLBI GO ESP, ExAC, Iranome, and our locally developed (GTAC) databases. It was not found in the literature, either. The six residues are inserted into a highly conserved region of ARSA, as evident through multiple-species alignment (Fig 1). The novel in-frame variant co-segregated with the disease in the family and was heterozygous in parents but homozygous in the patient (Fig 2). Given the PM2, PM4, PP3, PP4 ACMG/AMP criteria being fulfilled and the consistent phenotype, the c.109_126dup in *ARSA* was considered likely pathogenic in the patient.

### Molecular dynamics simulation

Molecular dynamics simulation method was employed to investigate the effect of the variant on the ARSA structure and function. To do this, 100-ns MD simulations were performed for WT-ARSA and mutant-ARSA to calculate all the required data. The obtained final structures of both ARSA proteins from 100-ns MD simulation were superimposed and are displayed in S1 Fig. No significant structural changes were found in the mutant-ARSA protein except in the loop shape region is composed of mutant residues Asp23, Leu24, Gly25, Cyc26, Tyr27, Gly28.

**Fig 3:**
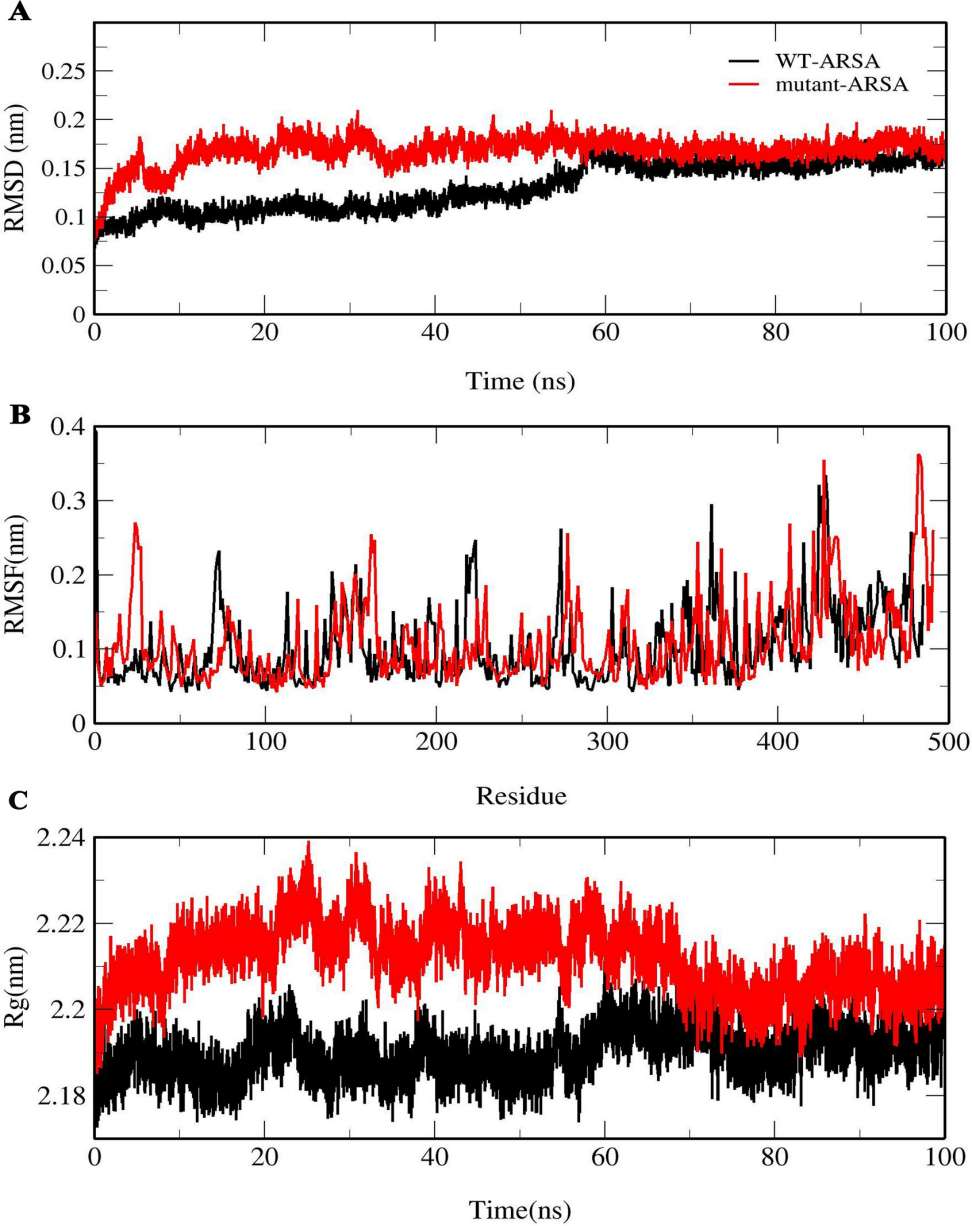
Conformational changes in ARSA proteins. (A) all-atom RMSD, (B) RMSF and (C) Rg of WT-ARSA and mutant-ARSA.

RMSD and RMSF were calculated for each production simulation. The average all-atom RMSD values for both ARSA proteins relative to the initial structures were calculated as 0.16 and 0.17 nm, respectively (Table 1 and Fig 3A).

**Table 1:** The average of various parameters during 100-ns MD simulation of wild type and mutant ARSAs.

Proteins	RMSD (nm)	Rg (nm)	Total SASA (nm2)	Active site SASA (nm2)	No. of Intramolecular HB	No. of protein-solvent HB
**WT**	0.130	2.18	189.41	0.075	337	799
**mutant**	0.167	2.21	197.12	0.125	336	833

Residual flexibility of both proteins was obtained and is displayed in Fig 3B. The RMSF graphs reveal that this variant significantly increased the fluctuation of residues in the loop shape regions, particularly the regions are composed of mutant residues in 23–28 position and residues in 480–485 position, and the regions with residues 160–163. In addition, the RMSF graphs show that the fluctuation of active site entrance residues of mutant-ARSA including Pro77, Gly78, Val79, His139, Gly158, Asp161, Gln162, Gly163, Tyr218, Thr274, Arg276, His393 are also increased, while the fluctuation of active site residues of mutant-ARSA was small.

The radius of gyration, which is defined as Rg, shows the compression and density of protein structure and stability, and the more compact a protein is, the more stable it will be [36] The average Rg values of WT-ARSA and mutant-ARSA were calculated as 2.18 and 2.21 nm, respectively (Table 1). The graphs of Rg data between both proteins are displayed in Fig 3C. The mutant-ARSA demonstrates a significant increment in Rg value.

**Fig 4:**
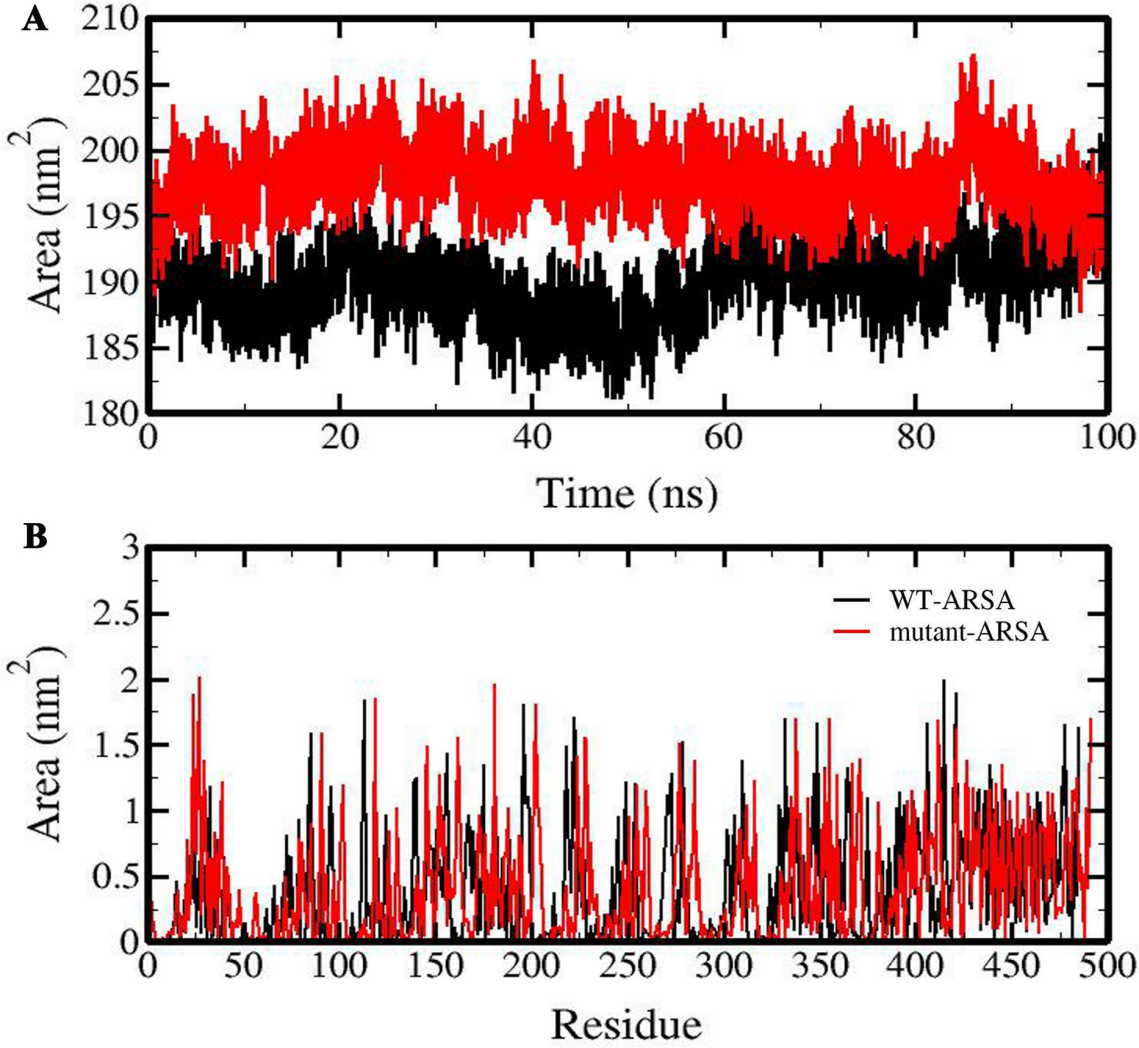
Graphical representation of solvent accessible surface area (SASA) of ARSA proteins. (A) Total SASA and (B) active site SASA of WT-ARSA and mutant-ARSA.

The hydrophobic core region of both ARSA proteins was investigated by calculating the total and residue solvent accessible surface area (SASA). Remarkable increment in the average of total SASA and relatively small increase in the average of active site SASA were obtained in the mutant-ARSA compared to WT-ARSA (Table 1 and Fig 4A). Among the active site residues of mutant-ARSA, residues Ala10, FGly57, Arg61, Lys111 and Asp269 with SASA of 0.02, 0.38, 0.061, 0.105 and 0.072 nm^2^ respectively indicate the most accessibility to solvent compared to those of in WT-ARSA with SASA of 0.002, 0.125, 0.011, 0.04, and 0.017nm^2^ respectively (Fig 4B).

**Fig 5:**
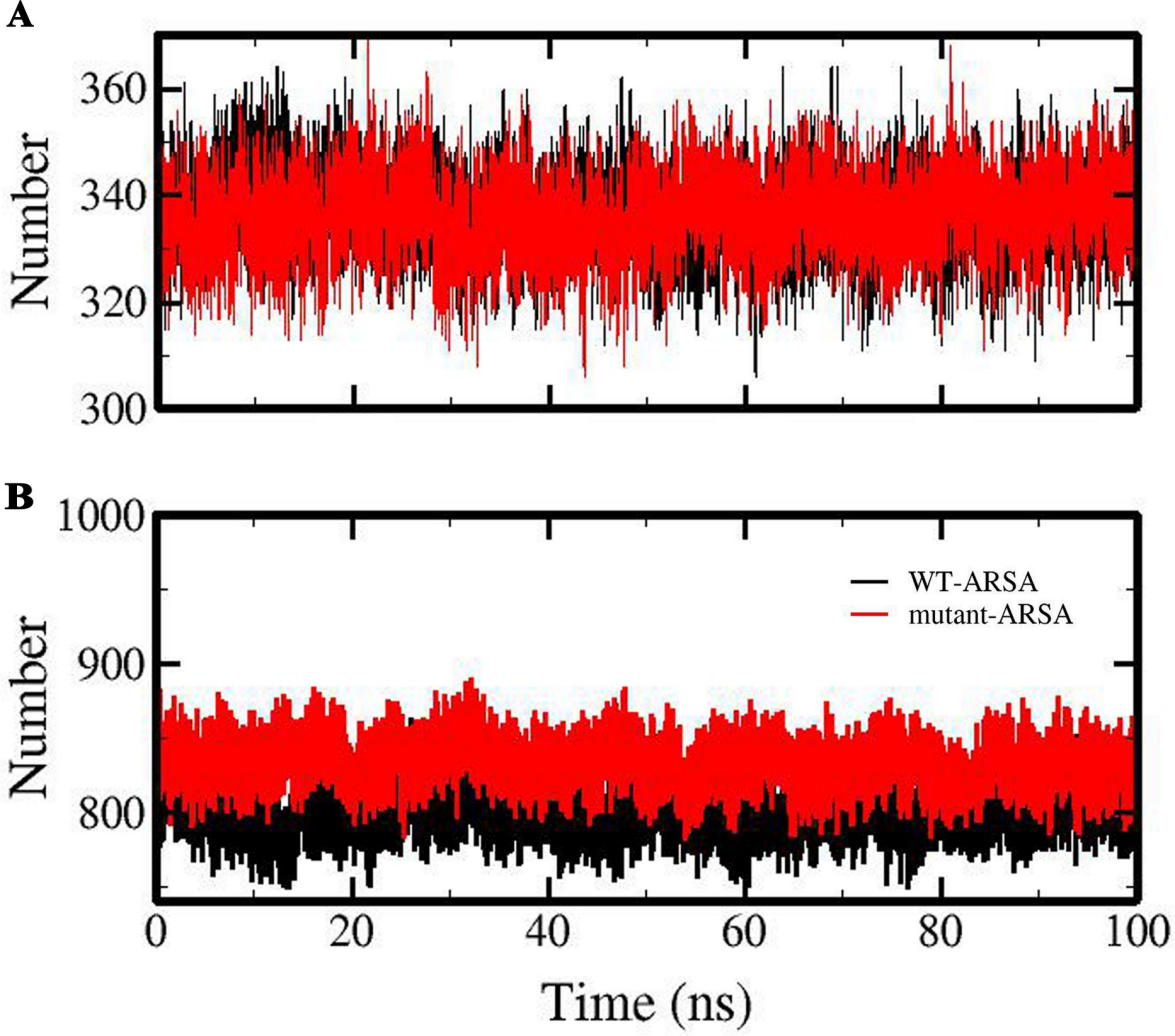
The pattern of hydrogen bonds during 100 ns MD simulation for ARSA proteins. (A) Intramolecular and (B) protein-solvent hydrogen bonds of WT-ARSA and mutant-ARSA.

Hydrogen bond (HB) analysis during MD simulation was investigated internally and the same analysis was conducted between the proteins and the solvent (protein-solvent) to understand the stability and the solubility of the ARSA proteins. The average number of intramolecular and protein-solvent HB networks and the patterns of them were obtained (Table 1 and Fig 5A and 5B).

As can be seen in Table 1 and Fig 5A, there is no substantial difference between the number of intramolecular HB of ARSA proteins, while a significant increment in the number of protein-solvent HB is observed in mutant-ARSA with an average of 833 HB compared to WT-ARSA with an average of 799 HB (Table 1 and Fig 5B).

The assignment of secondary structure elements is an essential component to investigate the structural behavior of protein. The variations in the secondary structure in WT-ARSA and mutant-ARSA were investigated using *do-dssp* function. The secondary structures of both proteins are almost similar and the six-amino acid insertion mutation is not able to induce any remarkable change in the secondary structure content because the mutation was in the loop region but not in a critical position for the secondary structure formation (S2A and S2B Fig) as illustrated in S1 Fig.

In order to calculate distances between all possible amino acid residues pairs of active site in both ARSA proteins, distances between Cα atoms of active site residues as a function of time were measured using gmx distance module. Eleven groups consist of Cα atoms of amino acid residues pairs of active site in WT-ARSA and mutant-ARSA were created and then were chosen to calculate the distances. These groups include Ala10—Asp11, Asp11—Asp12, Asp12—FGly51, FGly51—Arg55, Arg55—Lys105, Lys105—His107, His107—His211, His211—Asp263, Asp263—Asn264, Asn264—Lys284, Lys284—Ala10 in WT-ARSA and consist of Ala10—Asp11, Asp11—Asp12, Asp12—FGly57, FGly57—Arg61, Arg61—Lys111, Lys111—His113, His113—His217, His217—Asp269, Asp269—Asn270, Asn270—Lys290, Lys290—Ala10 in mutant-ARSA. As can be seen in distance plots in S3 Fig, the distances between almost all amino acid residues pairs in WT-ARSA are stable during 100-ns simulation while the groups of Asp12—FGly57, Arg61—Lys111, His217—Asp269 and Lys290—Ala10 in mutant-ARSA did not show stability in the course of 100-ns simulation.

**Fig 6:**
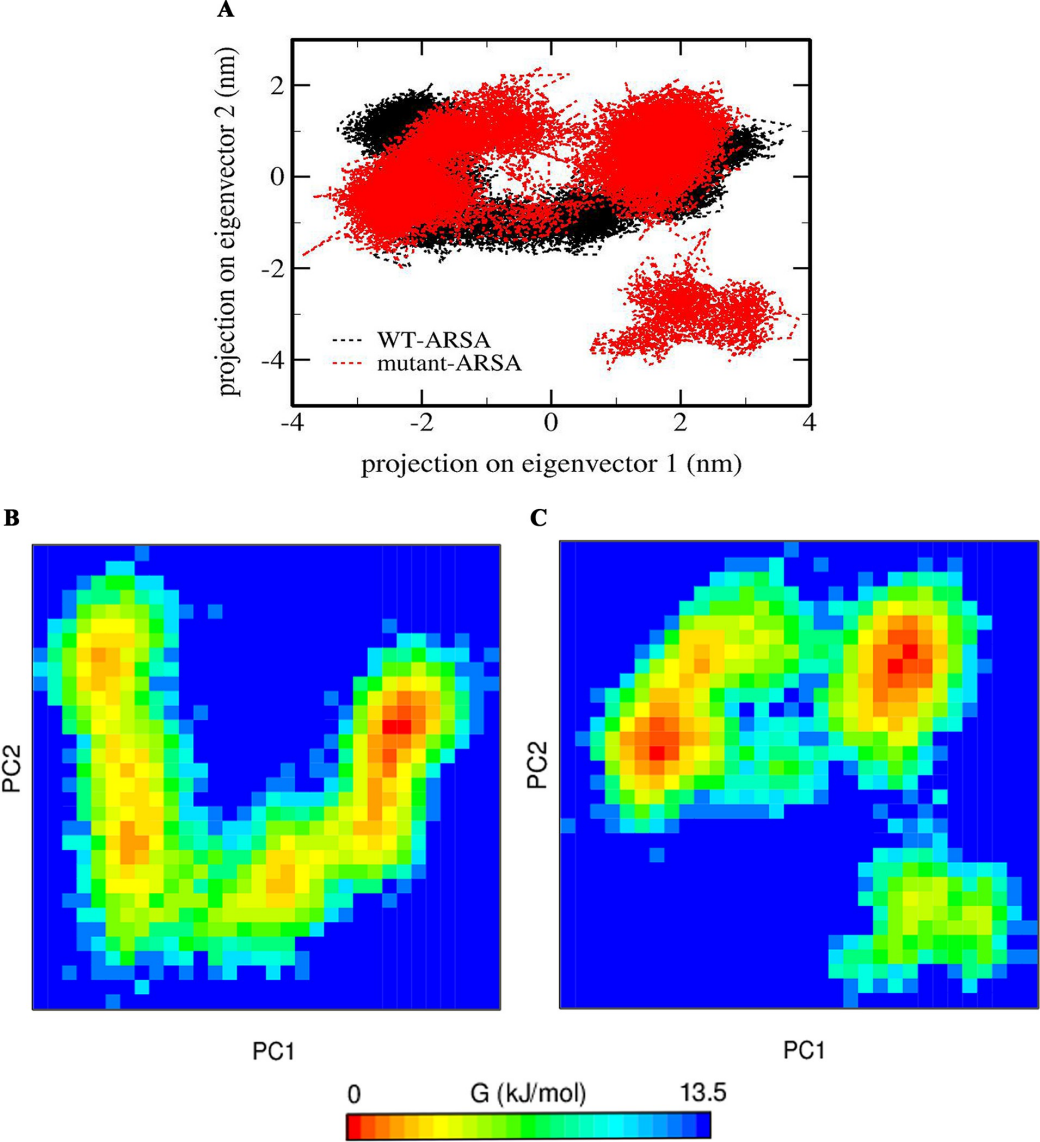
Conformational sampling and Free energy landscape (FEL) analysis of ARSA proteins. (A) Conformational sampling of WT-ARSA and mutant-ARSA proteins by 2D projection of the MD trajectory on PC1 and PC2. (B) FEL of WT-ARSA and (C) FEL of mutant-ARSA.

Principal Component Analysis (PCA) was performed to understand the dynamics of both ARSA proteins into a few principal motions, defined by eigenvalues and eigenvectors. The sampled conformations of WT-ARSA and mutant-ARSA in the essential subspace were calculated by projecting the protein backbone of the MD trajectory on eigenvectors 1 and 2 (Fig 6A). The projection of the PC1 and PC2 of a trajectory can represent a cluster of stable states in protein. As can be seen in Fig 6A, the mutant-ARSA displays a transition towards a more distant region of the phase spaces compared to WT-ARSA.

Free energy landscape (FEL) was calculated for two systems applying the first two PCs as reaction coordinates to find the conformational states of ARSA proteins. The FEL maps provide noticeable data on the diverse conformational states to the ARSAs in the 100 ns-MD simulation (Fig 6B and 6C). It is observed from Fig 6 that the PC1 and PC2 motion modes of the mutant-ARSA occupied larger spaces than those of the WT-ARSA, illustrating the conformational rearrangements which lie at the root of the mutation.

## Discussion

Owing to the rarity of MLD and the heterogeneity in its presentation, early diagnosis of MLD is challenging. To treat these individuals at an early stage of disease, accurate methods will be required to obtain an early diagnosis. Patients with MLD who get Haematopoietic Stem Cell Transplantation (HSCT) or gene therapy earlier in the disease have a better prognosis [11, 37, 38].

As a common molecular diagnostic tool, WES had a significant impact on identifying causative variations and determining the appropriate illness management strategy [39]. In the case of MLD, NGS can assist in illness identification, differentiating Pseudodeficiency diagnosis to avoid erroneous diagnoses based on ARSA activity, and identifying juvenile and adult variants of MLD to consider early treatment.

According to the Human Gene Mutation Database (HGMD) (http://www.hgmd.cf.ac.uk/ac/index.php), 303 *ARSA* mutations have been reported. Amongst Iranian MLD patients, c.931G>A(p.Gly311Ser) and c.465+1G>A variants are the most frequent alleles [40].

**Fig 7:**
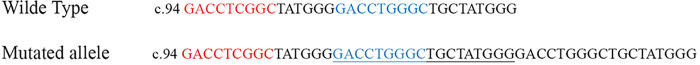
DNA sequences of Wilde Type *ARSA* and the mutated allele. The 5′ one is shown in red (GACCTCGGC) and the 3′ one is shown in blue (GACCTGGGC). The underlined sequence is duplicated in the mutated allele.

The structure of the DNA sequence around the 18-base-pair tandem duplication allele was investigated. In the normal sequence, we found a region with two short repeated sequences (Fig 7), which is indicative of a replication slippage. Polymerase slippage, that is expected to be as the possible cause of up to 75% of all indels, might explain the mechanism through which the mutation was generated [41]. This repeat, in our case is imperfect (GACCTCGGC and GACCTGGGC) (Fig 7), and the duplicated region is (GACCTGGGCTGCTATGGG). As a hypothesis, it appears polymerase slippage can cause this duplication at the genomic level, and subsequently leading to protein instability, and the resultant clinical manifestation in patients. We suggest further functional studies for interpreting the pathogenic mechanism of MLD.

In computational part of our study, the protein structure of the mutant-ARSA was constructed using Swiss Model server, then structural and functional effects of the six-amino acid insertion mutation in the ARSA protein were studied using 100 ns MD simulation to investigate the stability of mutant protein and the molecular mechanism of disease.

The RMSD plots of both proteins showed that they are constant during the simulation, with RMSD value of approximately 0.15 nm. The RMSF plots displayed that the most mutation effect on the fluctuation of residues was related to the loop shape regions and the active site entrance residues. It revealed that the regions with high fluctuation have a low contribution to creating contact with other amino acids [42], particularly the loop regions in positions 160–163, 480–485 and 23–28 with mutant residues. A comparison of Rg data of both proteins revealed that the Rg value of mutant*-*ARSA increased significantly, indicating a loss in compactness of the protein.

The investigation of the hydrophobic core region, a key parameter to assess protein folding and stability [43] in both proteins, displayed a noticeable increment in average total SASA and a small increase in average active site SASA in mutant-ARSA, demonstrating a large surface exposed to the solvent and this could be due to the solvent exposure of hydrophobic amino acids and subsequently influencing the protein folding [35, 43]. Furthermore, the HB analysis revealed that although there was no significant change in intramolecular HB of both proteins, the average number of protein-solvent HB in mutant-ARSA increased considerably, which indicates higher solubility than that of the WT-ARSA [44]. The insignificant reduction in the intramolecular HB formation in mutant- ARSA might be due to the fact that the variant occurs in a highly flexible loop at the protein surface and, subsequently it has minimal effect on the existing interactions [42, 45, 46], as clarified from the RMSF data. As elucidated from our Rg data as a measure of the protein stability, change in compression and size of mutant-ARSA could have been a plausible reason behind the changing in protein folding, increasing its solubility and following instability of mutant-ARSA [15]. Above all, the examination of the atomic distance between amino acid residues pairs of the active site in both proteins as a function of time demonstrated clearly that the mutation causes instability in mutant-ARSA active site, whereas the active site structure of WT-ARSA was stable in the course of simulation.

The most important parameters, principal component, and free energy landscape analysis were retrieved to investigate the global motion, folding, function, and, eventually stability of the protein [34, 47]. PCA demonstrated that mutant-ARSA covered a wide range of phase spaces compared to WT-ARSA. Given that the proteins perform their function via collective atomic motions and the stability of a protein is related to its collective atomic motion, PCA results indicate that the underlying reason for the impairment of the protein function might be given rise to an increase in the overall motion in mutant-ARSA. FEL analysis revealed that, compared to the WT-ARSA which displayed a single global energy minimum basin, mutant-ARSA illustrated wide global energy minima via a transition state which demonstrates the mutation caused the conformational rearrangement in protein. Indeed, the existence of several energy minima in the conformational space of mutant-ARSA proves the significant destabilization of the protein [47].

According to the *in silico* study, although no remarkable changes were observed in the overall structure, the secondary structure content and internal HB of both proteins, due to the position of the mutation in the flexible loop region far from the active site [45, 46, 48], changes in compactness, solvent accessibility, protein-solvent hydrogen bond, atomic distance measurement, protein motion, and FEL analysis obviously demonstrate the instability and dysfunction of the mutant-ARSA, which is in agreement with a remarkable reduction in ARSA Activity (0.066 mu/mg pro).

## Conclusion

In this paper, we report the successful application of WES for diagnosis and proper genetic counseling of MLD. The *in silico* data analysis of the ARSA protein provided an in-depth comprehension of destabilization and loss of conformational dynamics of the mutant-ARSA and elucidated clearly that the structure and function of mutant-ARSA are negatively influenced due to the mutation. The approach would be highly useful in pre-clinical studies for the discovery of novel therapeutic and management strategies for MLD.

## Supporting information

S1 Fig Schematic representation of obtained final structures of ARSA proteins after 100 ns MD simulation. (A) Superimposition of final structure WT-ARSA over mutant-ARSA. (B) Ribbon view showing the position of mutant amino acid residues. WT-ARSA and mutant-ARSA are shown in golden and green color respectively.
(JPG)

S2 Fig Secondary structure changes of ARSA proteins during 100 ns MD simulation. (A) WT-ARSA and (B) mutant-ARSA.
(JPG)

S3 Fig Distance calculation between Cα atoms of active site residues of WT-ARSA and mutant-ARSA as function of time (100 ns). Amino acid residues pairs of WT-ARSA active site are Ala10—Asp11, Asp11—Asp12, Asp12—FGL51, FGL51—Arg55, Arg55—Lys105, Lys105—His107, His107—His211, His211—Asp263, Asp263—Asn264, Asn264—Lys284, Lys284—Ala10 and those of mutant-ARSA are Ala10—Asp11, Asp11—Asp12, Asp12—FGL57, FGL57—Arg61, Arg61—Lys111, Lys111—His113, His113—His217, His217—Asp269, Asp269—Asn270, Asn270—Lys290, Lys290—Ala10.
(ZIP)
