# Supplementary figures and images for: Novel in-frame duplication variant characterization in late infantile metachromatic leukodystrophy using whole-exome sequencing and molecular dynamics simulation
Source: PLoS One. 2023 Feb 27;18(2):e0282304. doi: 10.1371/journal.pone.0282304 (PMC9970088; doi:10.1371/journal.pone.0282304)

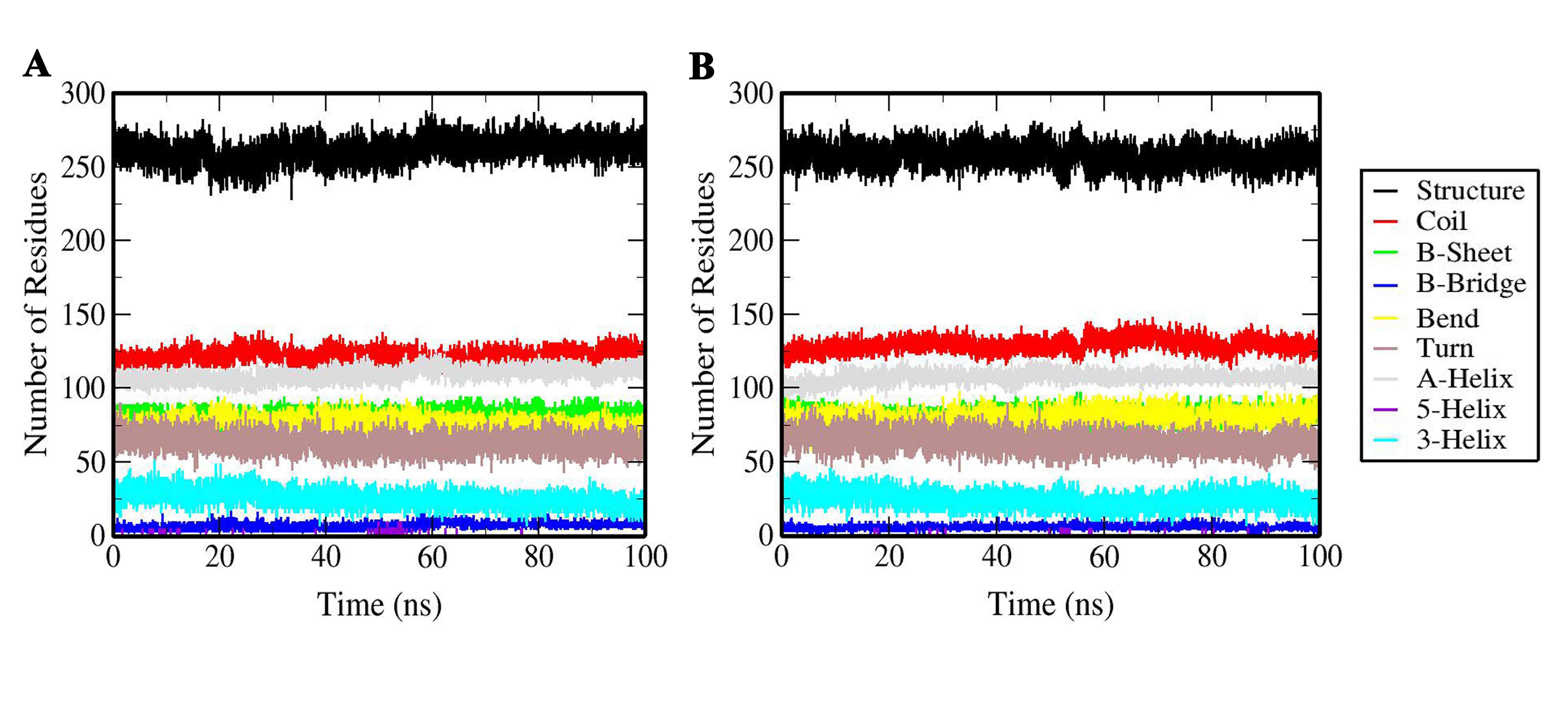

Supplement: S2 Fig — (A) WT-ARSA and (B) mutant-ARSA. (JPG) [file pone.0282304.s002.jpg]

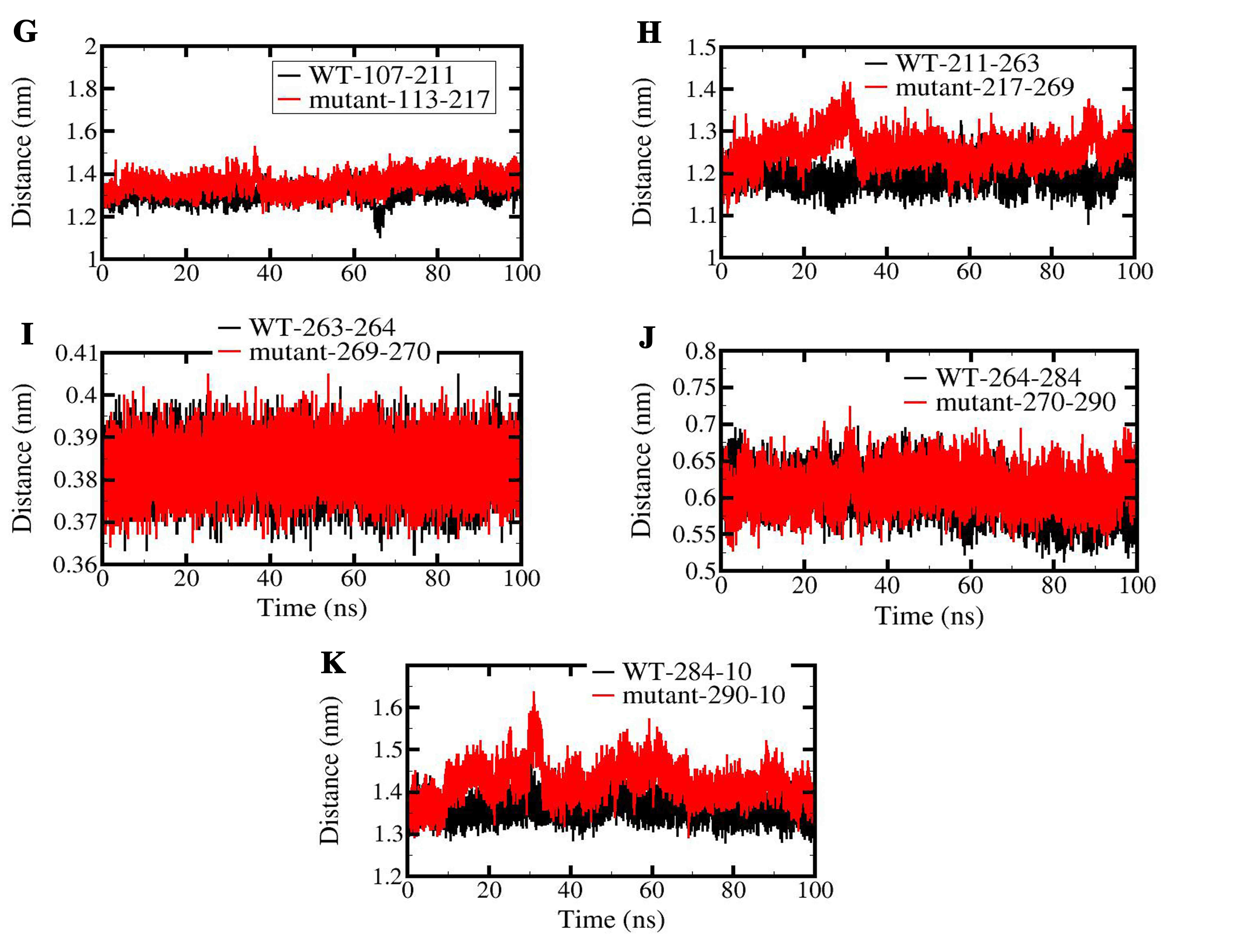

Supplement: S3 Fig — Amino acid residues pairs of WT-ARSA active site are Ala10—Asp11, Asp11—Asp12, Asp12—FGL51, FGL51—Arg55, Arg55—Lys105, Lys105—His107, His107—His211, His211—Asp263, Asp263—Asn264, Asn264—Lys284, Lys284—Ala10 and those of mutant-ARSA are Ala10—Asp11, Asp11—Asp12, Asp12—FGL57, FGL57—Arg61, Arg61—Lys111, Lys111—His113, His113—His217, His217—Asp269, Asp269—Asn270, Asn270—Lys290, Lys290—Ala10. (ZIP) [file pone.0282304.s003.zip › S3(2)_fig.jpg]
